# Süreyya-Dani technique, a new technique to address notching of soft triangle and opening external nasal valve

**DOI:** 10.3389/fsurg.2024.1385016

**Published:** 2024-06-14

**Authors:** Goran Latif Omer, Süreyya Şeneldir, Stefano Di Girolamo, Imad J. Habibullah, Avar F. Ahmed, Sahand S. Ali, Aland S. Abdullah, Ayman M. Mustafa, Berun A. Abdalla, Hemin Noori Hassan, Shvan H. Mohammed, Fahmi H. Kakamad

**Affiliations:** ^1^ College of Medicine, University of Sulaimani, Sulaymaniyah, Iraq; ^2^ Otorhinolaryngology, Head and Neck Department, Tor Vergata University, Rome, Italy; ^3^ Süreyya Şeneldir Clinic, ENT Department, American Hospital, Istanbul, Turkiye; ^4^ Scientific Affairs Department, Smart Health Tower, Sulaymaniyah, Iraq; ^5^ Department of Medicine, Shar Hospital, Sulaymaniyah, Iraq; ^6^ Kscien Organization for Scientific Research, Sulaymaniyah, Iraq; ^7^ College of Medicine, University of Garmian, Sulaymaniyah, Iraq; ^8^ Xzmat Polyclinic, Sulaymaniyah, Iraq

**Keywords:** rhinoplasty, Süreyya-Dani technique, aesthetic rhinoplasty, functional rhinoplasty, nasal valve

## Abstract

**Introduction:**

While different methods are employed for fixing narrowed nasal valves and preventing the notching of soft triangles, this study aims to demonstrate the effectiveness of a new technique called Süreyya-Dani Technique.

**Methods:**

This prospective study composed 100 patients who underwent rhinoplasty using the Süreyya-Dani technique. All patients presented with either notching of the soft triangle and/or external nasal valve dysfunction. Patients with the absence of soft triangle notching and external nasal valve dysfunction were excluded from this study. Facial analysis was conducted for all patients to identify any asymmetry in the face, and all nasal defects were identified. Descriptive statistics were calculated for different variables. Analytical statistics, namely Chi-Square test, was conducted with a significance level set at *P* < 0.05.

**Results:**

In the current study, 100 patients were involved, out of which 63 (63%) were female and 37 (37%) were male. The participants' ages ranged from 18 to 46 years, with a mean age of 30 years. various chief complaints were found among patients, with the majority 37(37%) expressing cosmetic concerns. A statistically significant difference was found for the association of nasal tip defects with genders, intraoperative findings, and chef complaints, and the association between the degree of external valve insufficiency and crural weakness (*P*-value < 0.05).

**Conclusion:**

Despite many techniques that have been put forward to fix narrowed nasal valves and prevent notching of the soft triangle, the Süreyya-Dani Technique could work to prevent its occurrence successfully.

## Introduction

Rhinoplasty is a form of plastic surgery that alters the shape or appearance of the nose while maintaining or improving the nasal airway. The main reason for the operation could be either cosmetic, functional, or both, and it could involve additional surgical procedures on the nasal valve, nasal septum, nasal turbinates, or paranasal sinuses (1, 2). According to the American Society of Plastic Surgeons, rhinoplasty ranks as the fifth most sought-after procedure in cosmetic surgery (2).

Even though nasal anatomy is simple surgically, aggressive and hurried surgery that disregards anatomical considerations can result in irreversible side effects (3). Among the various anatomic irregularities that individuals undergoing rhinoplasty may experience, four types are typically significant and common. Failure by surgeons to address these can predict unfavorable postoperative outcomes. These are middle vault collapse, articular cartilage malposition, insufficient tip projection, and a low radix (4).

Although there are continuous innovations in rhinoplasty techniques, the procedure still has its complications, one of which is nasal valve dysfunction. According to genuine literature regarding facial plastic surgery, the prevalence of nasal valve dysfunction is approximately 13%. In up to 95% of cases, the nasal valve has been implicated as the cause of persistent nasal obstruction after septorhinoplasty. The primary reason is typically a previous rhinoplasty, with other contributing factors being various surgical interventions, trauma, facial paralysis, congenital issues, and the natural aging process. One instance of nasal valve compromise during surgery can occur due to accidental damage to the soft triangle, which represents the space between the nasal dome and the rim of the nostril (5, 6). The collapse of the nasal valve impairs nasal breathing, which significantly degrades the quality of life (7). The collapse of the external nasal valve, in particular, may result from the previously mentioned factors, specifically alar base misalignment, scarring in the valve region or nasal vestibule after surgery, caudal septal deviations, or drooping of the nasal tip (8). Nasal valve insufficiency can cause unpleasant symptoms such as blockage, inability to breathe fully via the nose, and stuffiness, and surgery may be required for severely symptomatic patients (9).

Various methods can be employed to fix a narrowed nasal valve, such as alar strut grafts, alar batten grafts, Z-plasty, lateral crus pull-ups, skin grafts, or composite grafts (8). The current study aims to demonstrate the effectiveness of a current method for averting notching in the soft triangle, maintaining the integrity of the external nasal valve, and correcting irregularities or asymmetry of the upper angle of the nostrils with a graft named the Süreyya-Dani Technique.

## Materials and methods

### Patient population and eligibility criteria

This prospective study comprised 100 patients who underwent rhinoplasty with the Sürreye-Dani technique at the Ear, Nose, and Throat Department of the Royal Hospital. The patients ranged in age from 18 to 46 years old, with a mean age of 30, and there was no gender preference. The data were gathered during the patient's visits to the clinic and perioperatively. All patients presented with unilateral or bilateral notching of the soft triangle and/or external nasal valve dysfunction.

All patients included in the study underwent a comprehensive clinical examination and consultation, during which the shape of the nose was discussed. A facial analysis was conducted for all patients to identify any asymmetry in the face. All nasal defects were identified and discussed with the patient, and the necessary steps to achieve a natural-looking nose were determined pre- and intraoperatively. Pre-operative photographs were taken in the clinic studio, including seven views: front, head up, head down, oblique right, oblique left, left profile, and right profile. Intraoperative photographs were taken both following intubation and after the operation as well. All data were obtained by an Ear, Nose, and Throat specialist at the private clinic with the patient's consent, and all operations were performed in the Royal Hospital's Ear, Nose, and Throat Department. Patients with the absence of soft triangle notching and external nasal valve dysfunction were excluded from this study since they would not need the Süreyya-Dani technique otherwise.

### Surgical intervention

After ensuring their fitness for anesthesia, the patients were placed under general anesthesia and prepared for surgery in the reverse Trendelenburg position. The surgical procedure commenced with the injection of 5.4 ml of lidocaine-epinephrine solution (Persocaine-E) into the mucoperichondrium, dorsal skin, nasal floor, and nasal septum. An inverted v-shaped incision was then made in the columella at its narrowest point, followed by dissection of the lower lateral cartilage, dorsum of the nose, and upper lateral cartilage. Subsequently, rasping with de-humping was performed, followed by lower lateral cartilage trimming and septoplasty. Lateral spreader grafts and columellar strut grafts were then placed, along with dome sutures and lateral crural grafts. A cup graft was also utilized, and the middle crus of the lower lateral cartilage was assessed, as weakness at this part of the cartilage is the most significant risk factor for nostril asymmetry, soft triangle notching, and closure of the external nasal valve. If there was weakness in this part, it was corrected by the vertical alar resection technique or an adnexal underlay graft, and the skin was closed. The septum was quilted, and preoperative and intraoperative assessments were conducted to determine the need for a Süreyya-Dani technique. In some cases, the notch was corrected with the use of the previous techniques during surgery (including lateral crural grafts, cup grafts, and/or adnexal grafts), eliminating the need for a Süreyya-Dani technique. However, when the other techniques were not sufficient, or in certain instances, such as during the correction of a crooked nose, the notch became apparent, and a graft was placed for the Süreyya-Dani technique.

Due to its importance in nasal breathing, any defect in the external nasal valve results in nasal obstruction. Since the nasal valve is made up of the lateral crus of the lower lateral cartilage, the septum, and the skin of the nasal vestibule, any pinching of the soft triangle will therefore result in a collapse of the skin over the lower lateral cartilage. This skin collapse will consequently result in the lower lateral cartilage itself collapsing over the portion of the nasal septum that contributes to the formation of the external nasal valve. Therefore, a Sürreya-Dani graft is used to hold the skin and prevent it from notching over the lower lateral cartilage, subsequently preventing the collapse of the lower lateral cartilage on the septum and hence, the narrowing of the external nasal valve.

**Figure 1:**
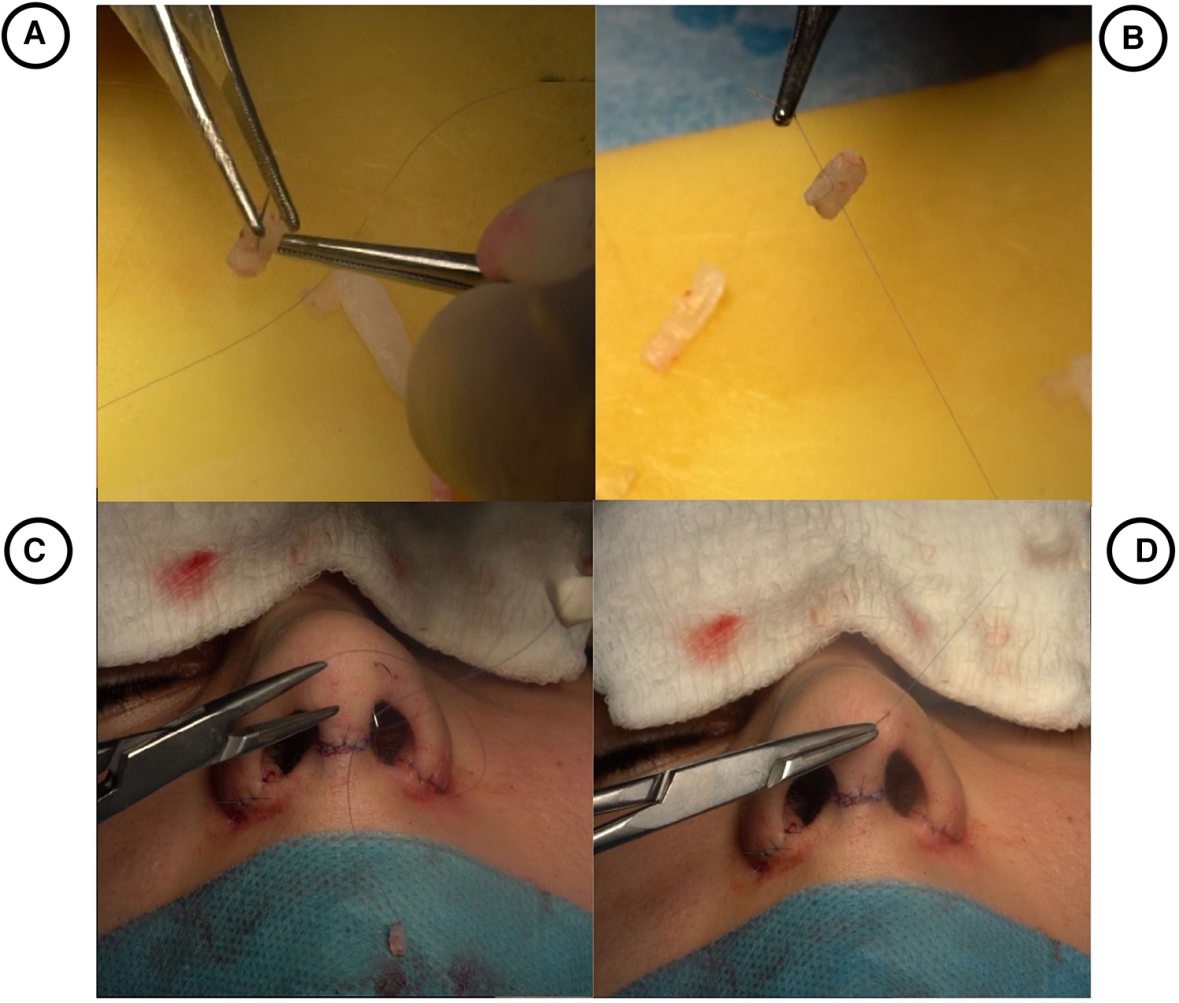
(**A**) preparation of Süreyya-Dani graft according to the size of the defect. (**B**) Stitching the graft with 6.0 PDS. (**C**) Positioning the graft inside the pocket of the defect. (**D**) Tightening the suture in the soft triangle.

The graft, measuring 4–5 mm in width, 5–6 mm in length, and 2–3 mm in thickness, was harvested from either the septum or lateral crus of the lower lateral cartilage. For patients with a soft triangle defect, a protractor was used preoperatively to measure the defect and the cartilage was sculpted accordingly. A 6.0-round needle PDS suture was stitched to the Süreyya-Dani Graft and was inserted into the soft triangle through the alar rim incision that was initially performed. It was then taken out of the defect site. subsequently, the suture was pulled, thereby stabilizing the graft within the defect. Moreover, the suture is then stitched to the graft itself to provide further stabilization (Figure 1). This stitch was removed after three weeks of the follow-up period.

**Figure 2:**
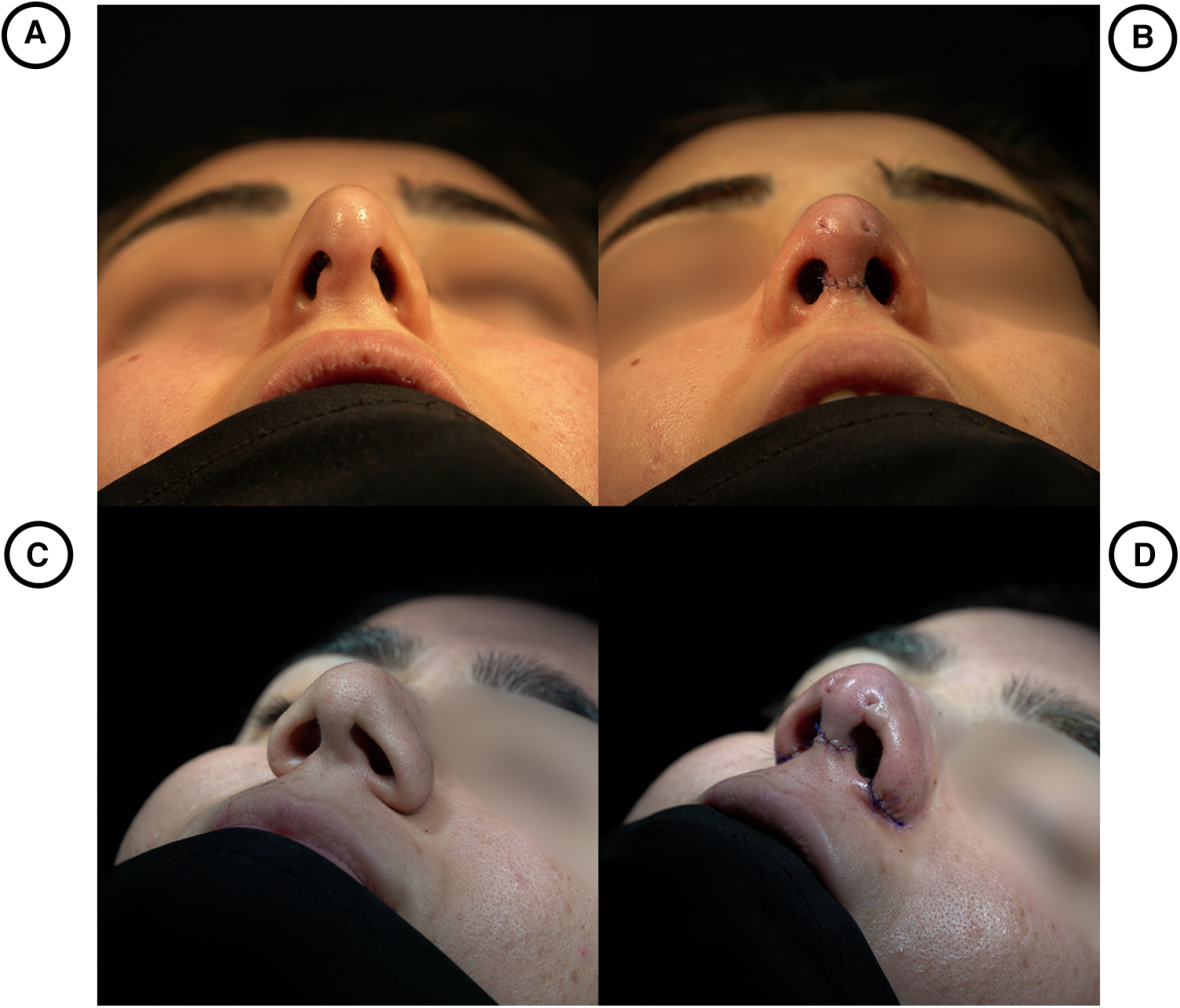
(**A**) (Basal view; thin skin) and (**C**) (oblique profile view; thick skin) show preoperative views of the soft triangle defect with middle crural weakness. (**B**) (Basal view; Thin Skin) and (**D**) (Oblique Profile view; Thick Skin) are postoperative views with the Süreyya-Danni graft in position and tightened with 6.0 PDS.

Each patient was followed up in the 1st week, 2nd week, 3rd week, 1st month, 3rd month, and finally, a year post-operation (Figure 2).

### Clinical measures of disease severity

**Figure 3:**
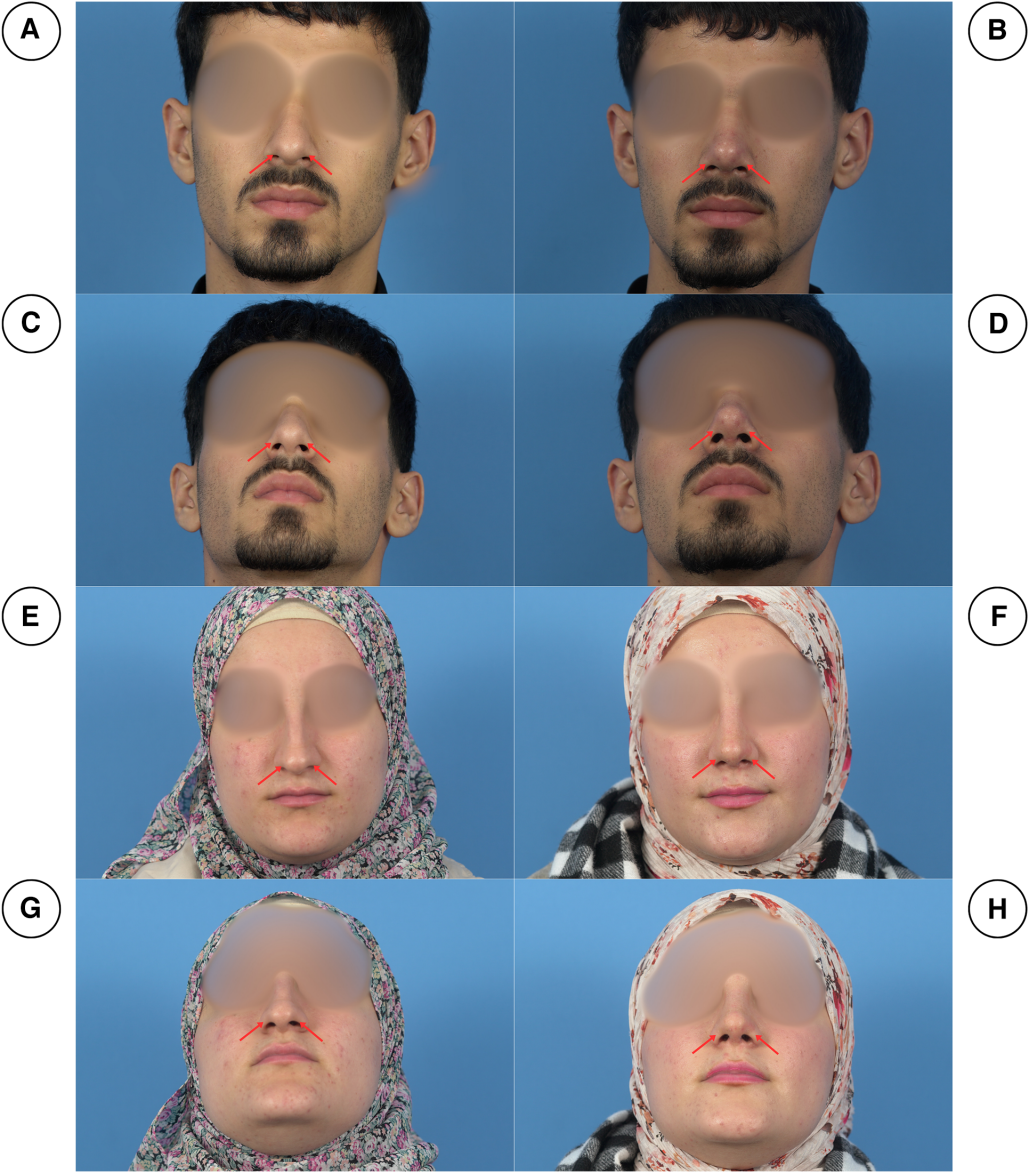
Pre-operation (right column) and post-operation (left column) pictures of two patients in frontal views (**A, B, E, F**) and basal views (**C, D, G, H**). As can be seen, the patients had notching of the soft triangles (red arrows), which was corrected postoperatively.

**Figure 4:**
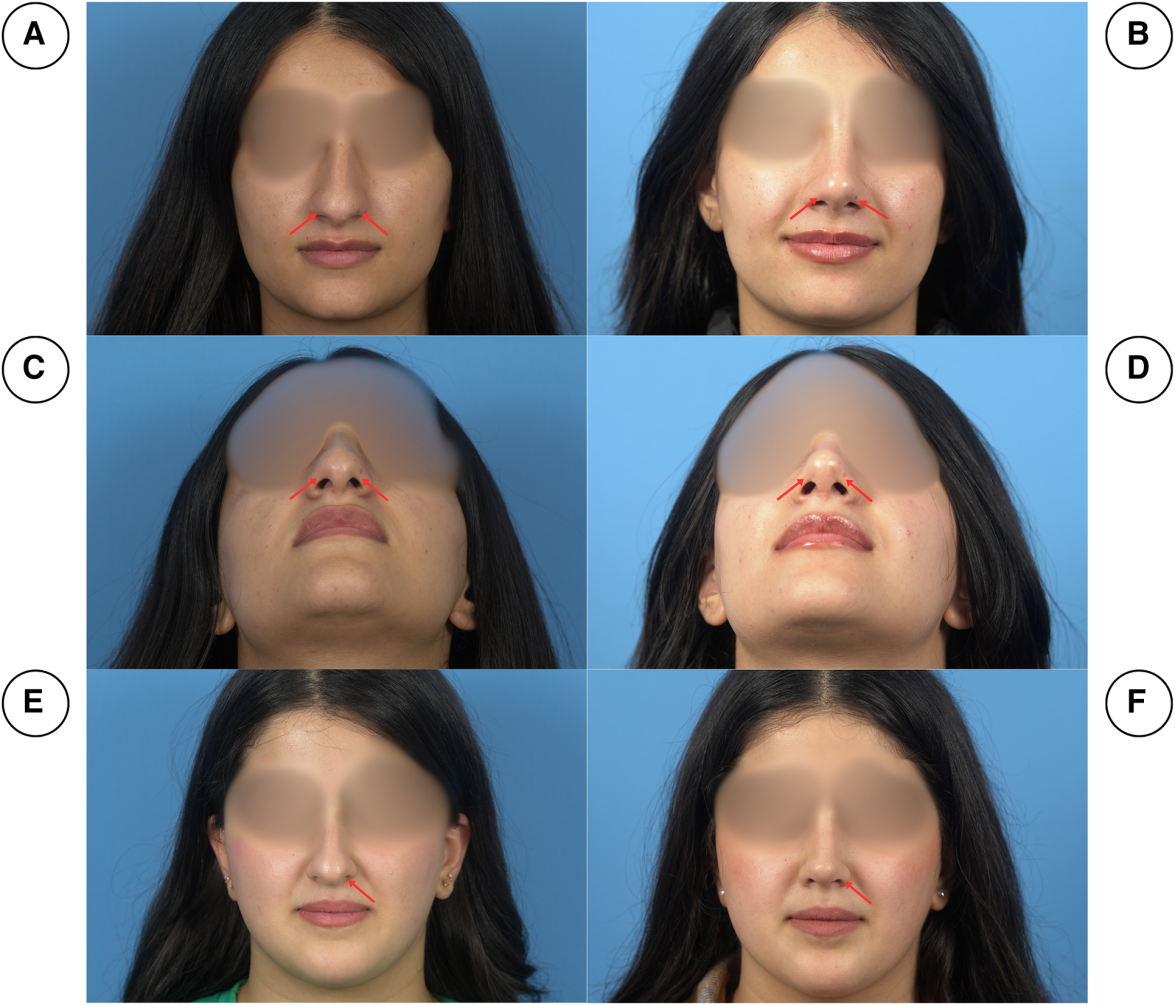
Pre-operation (right column) and post-operation (left column) pictures of two patients in frontal views (**A, B, E, F**) and basal views (**C, D**). As can be seen, in (**A**) and (**C**), the patient has bilateral notching of the soft triangle, however, the patient in (**E**) has unilateral left-sided notching. Postoperatively (**B, D, F**), the notching was corrected.

Due to the nature of the procedure, disease severity was analyzed through a history of respiration problems and clinical examinations. During examination, a meticulous facial analysis was done, which included assessing the ratio between nasal length and tip projection (about 0.67), checking the nasofrontal (115–130°), nasolabial (90–95° for males and 95–115° for females), nasofrontal (30–40°) and nasomental angles (142–152°) and the alar base width (31–33 mm), radix analysis and examining the quality of skin (10–13). Additionally, the aesthetic outcomes were further scrutinized and recorded through perioperative documentation, as all patients were photographed preoperatively, instantly after the operation, and throughout the follow-up period (1 week, 2 weeks, and 3 weeks after the operation) (Figures 3, 4).

### Nasal function assessment

Nasal function was evaluated pre- and postoperatively in all patients through the implementation of the Cold Spatula test, Cotton test, and Cottle's test (14–16). Mist formation on the cold spatula was considered a negative finding, while the absence of mist due to obstruction was regarded as a positive finding. In Cotton test, Cotton movement was regarded as a negative finding, while the absence of cotton movement due to obstruction was considered a positive finding. In the assessment by Cottle test, ease in breathing with the cheek lifted upwards and laterally (resulting in an increased internal nasal valve angle) was considered a positive finding. Conversely, when breathing remained similar before and after lifting the cheek, the finding was regarded as negative.

### Reported outcome

The study employed the Rhinoplasty Outcome Evaluation (17), consisting of six questions posed to the patients. These questions assessed various aspects, including the liking of nose appearance, nasal breathing ability, perception of others' opinion on nose, impact of nasal appearance on social or professional activities, confidence in current nasal appearance, and desire for surgical alteration of nose appearance or function.

### Statistical analysis

In this study, descriptive statistics, such as mean, were found for some variables, including the age distribution of the patients. Analytical statistics, namely Chi-Square, were found for the variables, and the *P*-value was set to be less than 0.05.

## Results

Of the total patients enrolled in this study, 63% were female and 37% were female. Furthermore, young patients seemed to be the most common age group with the 18–24 and 25–34 age group together making up close to 70% of the participants. Patients had different presenting chief complaints, with the majority of them 37% presenting with cosmetic concerns that were notching. Further characteristics of the enrolled participants are given in Table 1.

**Table 1:** Demographic and clinical characteristics of participants.

Variables	Frequency	Percentage (%)
Gender		
Male	37	37
Female	63	63
Age		
18–24	32	32
25–34	36	36
35–44	27	27
45–54	5	5
Nasal tip defect		
Notching of soft triangle	39	39
Tip drop	41	41
Overhanging of the columella	20	20
Chief complain		
Cosmetic (Notching)	37	37
Functional (External valve)	33	33
Both	30	30
External valve insufficiency degree	22	22
Total		
Partial	39	39
None	39	39

In this study, regarding the association between the gender of participants and nasal tip defect, overhanging of the columella was more common in males constituting 60% of the total, while tip drop was more common among females comprising 73.17% of total with a *P*-value <0.041) (Table 2).

**Table 2:** Association of nasal tip defect with gender of patients.

Variable	Nasal tip defect			
	Notching of soft triangle	Tip drop	Overhanging of the columella	*P*-value
Gender				
Male	14 (35.9%)	11 (26.8%)	12 (60%)	0.041
Female	25 (64.1%)	30 (73.2%)	8 (40%)	

Accordingly, the association between intraoperative findings and chief complaints showed a statistically significance difference among participants (*P*-value = 0.003) (Table 3).

**Table 3:** Relationship between intraoperative findings and chef complaint.

Variable	Intraoperative findings				
	Thin skin	Middle crus weakness	Lateral crus weakness	Thick skin	*P*-value
Chief complain					
Cosmetic (Notching)	5 (17.8%)	16 (48.5%)	6 (28.6%)	10 (55.6%)	0.003
Functional	11 (39.3%)	7 (21.2%)	13 (61.9%)	2 (11.1%)	
Both	12 (42.7%)	10 (30.3%)	2 (9.5%)	6 (33.3%)	

Statistical analysis showed that external Valve Insufficiency Degree is associated with different crural weaknesses. The Middle and Lateral crural weakness was the most common type of crural weakness in both the total (63.63%) and partial external valve insufficiency patients (35.9%), while medial is the most common type of weakness among those with no degree of external valve insufficiency constituting 35.89% of total (*P*-value = 0.023) (Table 4).

**Table 4:** Association between degree of external valve insufficiency and crural weakness.

Variable	External valve insufficiency degree			
	Total	Partial	None	*P*-Value
Crural weakness				
Middle and lateral crura	14 (63.6%)	14 (35.9%)	10 (25.6%)	0.023
Medial	3 (13.6%)	11 (28.2%)	14 (35.9%)	
Both	4 (18.2%)	11 (28.2%)	6 (15.4%)	
None	1 (4.5%)	3 (7.7%)	9 (23.1%)	

The evaluation of nasal function in 100 patients demonstrated that 36% had a negative preoperative cold spatula test. Among those with positive preoperative tests on the right and left sides (40% and 24%, respectively), only 2% tested positive postoperatively on the right side, with an additional 3% of patients initially negative for the test showing positive results postoperatively on the right side. Approximately similar results were obtained using the Cotton and Cottle tests (Table 5). Rhinoplasty outcome evaluation, revealed that approximately 95% of patients expressed satisfaction with their nasal appearance, reported no limitations in social or professional activities due to nasal appearance. Additionally, a majority of patients (90%) reported no interest in surgically altering their nose (Table 6).

**Table 5:** Nasal function assessment by cold spatula, cotton, and cottle test.

Variables		Postoperative test (cold spatula, Cotton, Cottle)	
		Negative	Positive right side
Preoperative cold spatula test	Negative	33 (34.7%)	3 (60%)
	Positive Rt side	39 (41.1%)	1 (20%)
	Positive Lt side	23 (24.2%)	1 (20%)
	Total	95 (100%)	5 (100%)
Preoperative Cotton’s test	Negative	33 (34.7%)	3 (60%)
	Positive Rt side	39 (41.1%)	1 (20%)
	Positive Lt side	23 (24.2%)	1 (20%)
	Total	95 (100%)	5 (100%)
Preoperative Cottle’s test	Negative	34 (35.85%)	3 (60%)
	Positive Rt side	42 (44.2%)	1 (20%)
	Positive Lt side	19 (20%)	1 (20%)
	Total	95 (100%)	5 (100%)

**Table 6:** Responses to rhinoplasty outcome evaluation questions.

Outcome	Frequency (%)
Do you like appearance of nose?	
Not at all	0 (0.0)
Somewhat	0 (0.0)
Moderately	5 (5)
Very much	28 (28)
Completely	67 (67)
Ability to Nasal Breathing?	
Not at all	0 (0.0)
Somewhat	1 (1)
Moderately	6 (6)
Very much	23 (23)
Completely	70 (70)
How do your loved ones view your nose?	
Not at all	0 (0.0)
Somewhat	0 (0.0)
Moderately	0 (0.0)
Very much	20 (20)
Completely	80 (80)
Your nasal appearance limits you social or professional activities	
Always	0 (0.0)
Usually	0 (0.0)
Sometimes	0 (0.0)
Rarely	0 (0.0)
Never	100 (100)
Confident in nasal appearance	
Not at all	0 (0.0)
Somewhat	0 (0.0)
Moderately	0 (0.0)
Very much	24 (24)
Completely	76 (76)
Interest in nose surgery?	
Definitely	0 (0.0)
Most likely	0 (0.0)
Possibly	10 (10)
Probably not	25 (25)
No	65 (65)

## Discussion

Rhinoplasty is one of the most commonly done surgeries, and though it has many indications, types, and techniques, it also has its complications. The complications can also be divided into cosmetic or functional, but they are interrelated, as many cosmetic deformities can lead to functional disturbances. An example of this is soft triangle notching. In this delicate triangular area, skin is in contact with skin with no subcutaneous tissue or cartilage in between, thus making it amenable for injuries during intranasal incisions, leading to notching. Besides the unaesthetic appearance of the notches, they cause dysfunction of the external nasal valves, which is of high significance (18). In 1994, Constantian demonstrated the functional significance of nasal valves, particularly the external nasal valve, when he evaluated the separate reconstruction of external and internal nasal valves with preoperative and postoperative rhinomanometry and observed a 2.6 and 2.0-fold increase in nasal airflow, respectively. When he combined the treatment of septal deviation with the reconstruction of the external and internal nasal valves, nasal airflow increased by a factor of 4.9. Each of these outcomes was statistically significant (19). The literature underscores the necessity of utilizing objective metrics to thoroughly assess outcomes in functional rhinoplasty. A study by Paul et al. focused on this aspect, concentrating on functional rhinoplasty involving spreader grafting and employing acoustic rhinomanometry alongside validated outcome measurements. Their findings emphasized the crucial role of objective evaluations in determining the efficacy of surgical interventions aimed at enhancing nasal function. This cohort study provides valuable insights into the domain of functional rhinoplasty, reinforcing the functional implications of nasal deformities and the significance of surgical approaches in reinstating nasal airflow dynamics (20).

When the external nasal valve is collapsed, the only permanent treatment is surgery. One of the techniques is marginal incision, where an incision is made in the caudal margin of the lower lateral cartilage to make a pocket by dissecting the alar facial groove and placing a graft in that pocket. The grafts that are put in the “pockets” are from either the concha cavum or the nasal septum, after cutting them into the appropriate dimensions (21).

Regarding techniques used to fix soft triangle defects, according to a study, Z-plasties are an option that utilizes local tissue for the reconstruction, and they concluded that the small procedure results in color-matched, thin, and aesthetically acceptable results. However, the Z-Plasty technique can only be used for small deformities (22). Besides correcting deformities, many studies have tried to figure out ways to prevent soft triangle defects in the first place. For example, according to a study by Campbell et al., 5 main interventions should be accomplished to prevent such defects: accurate dissection and incisions, closing dead spaces, avoiding unwanted tension during closure, cartilage internal support, if needed, and finally, external support postoperatively (23). In this study, another technique is described, which follows all 5 interventions and is used to prevent soft triangle notching, thus preventing external nasal valve dysfunction, called the Süreyya-Dani technique. Through this technique, patients undergoing septorhinoplasty are evaluated pre- and intraoperatively based on whether or not they are liable to have notching of the soft triangle postoperatively. Preoperatively, if the patient has very thin or very thick skin, or if they already have notching of the soft triangle, the graft will be added. Another preoperative risk factor is having an exaggerated amount of dead space in the soft triangle area, which can be assessed through physical examination when there is soft triangle notching with a slight press on the tip. Furthermore, the graft will also be added if the patient is found to have a weak middle cru of the lower lateral cartilages.

Numerous causes and risk factors for notching were identified among patients in this study, and upon statistical analysis, many exhibited significant statistical correlations. For example, according to the current study, most patients with a weak lateral cru had some degree of external nasal valve dysfunction, total or partial. This finding is consistent with other studies, including Menger's, which states that the most common cause of external nasal valve dysfunction is either a confined nasal vestibule or a “floppy” lateral crura (24). The current study also demonstrates that female patients are more likely to present with soft triangle notching and that soft triangle notching is most likely to happen in patients with thin-skinned noses preoperatively, that is if no preventative measures are taken intraoperatively. This funding goes parallel with that of many studies, such as those done by De Almeida or Friedman, with the latter later explaining that excessively thin skin cannot provide the nasal sidewalls with the integrity they need to withstand collapse upon the negative pressure of inhalation (25).

One limitation of the current study is the absence of a control group, which prevents a comprehensive understanding of the significance of individual variables. However, this is unacceptable in our society, as patients expect preventive measures for potential disorders. Therefore, it is recommended to employ these methodologies in multi-center studies in which a broader and more diverse population is involved to better evaluate outcomes. Longer-term follow-up would be valuable to assess the durability and long-term outcomes of the Süreyya-Dani technique, including potential complications or recurrence of nasal valve dysfunction.

## Conclusion

While various methods have been proposed to address a narrowed nasal valve and prevent notching of the soft triangle, the Süreyya-Dani Technique serves as a new and innovative approach to fix this issue. Further research is necessary to confirm the findings reported in this study.

## Data Availability

The original contributions presented in the study are included in the article/Supplementary Material, further inquiries can be directed to the corresponding author.
